# Successful Therapeutic Plasma Exchange in Refractory Anti-MDA5-Positive Dermatomyositis and Rapidly Progressive-Interstitial Lung Disease: A Case Report

**DOI:** 10.4274/TJAR.2026.252250

**Published:** 2026-08-28

**Authors:** Bedih Balkan, Ebru Kaya

**Affiliations:** 1 University of Health Sciences Türkiye Kanuni Sultan Süleyman Training and Research Hospital, Clinic of Anaesthesiology and Reanimation, Intensive Care Unit, İstanbul, Türkiye

**Keywords:** Anti-MDA5, dermatomyositis, RP-ILD, TPE

## Abstract

Anti-MDA5-positive clinically amyopathic dermatomyositis (CADM) is closely linked to rapidly progressive-interstitial lung disease (RP-ILD) and is associated with high mortality rates. This report discusses a 60-year-old female patient diagnosed with anti-MDA5-positive CADM who developed RP-ILD that was resistant to pulse steroid therapy, tacrolimus, and intravenous immunoglobulin. As her respiratory condition worsened alongside rising inflammatory markers, therapeutic plasma exchange (TPE) was initiated. Following four sessions of TPE administered on alternate days, significant clinical and laboratory improvements were observed. This case underscores the potential life-saving impact of early TPE for patients with anti-MDA5-positive CADM and RP-ILD who do not respond to traditional immunosuppressive treatments.

**Main Points**

• Anti-MDA5-positive dermatomyositis may present as an amyopathic condition, but poses a significant risk of developing rapidly progressive-interstitial lung disease, which carries a high mortality.

• Standard treatments, such as high-dose corticosteroids, calcineurin inhibitors, and intravenous immunoglobulin, often fail to halt neurological deterioration, as in this patient.

• Therapeutic plasma exchange (TPE) can effectively remove circulating anti-MDA5 antibodies and proinflammatory mediators, leading to substantial clinical and laboratory improvements.

• In this instance, TPE resulted in marked reductions in ferritin and C-reactive protein levels and in improved oxygenation and radiological outcomes shortly after the initial treatment.

## Introduction

Anti-MDA5-positive clinically amyopathic dermatomyositis (CADM) is a rare variant of idiopathic inflammatory myopathies, distinguished by specific skin manifestations and minimal or absent muscle involvement. The principal clinical challenge arises from the development of rapidly progressive-interstitial lung disease (RP-ILD), which stands as the foremost cause of morbidity and mortality in affected individuals.^1, 2^ Reported mortality rates exceed 50% within six months post-diagnosis, highlighting the aggressive nature of this condition.^3, 4^ Current management approaches include administering high-dose corticosteroids along with calcineurin inhibitors or cyclophosphamide.^2, 3, 4^ Nevertheless, many patients exhibit inadequate responses to aggressive immunosuppression and subsequently experience rapid clinical decline; thus, early identification of patients at high-risk is essential. Various laboratory indicators, including elevated serum ferritin levels, lactate dehydrogenase (LDH), and systemic inflammatory markers, have been correlated with disease severity and a poor prognosis.^5, 6^ These observations imply that ongoing inflammation and lung injury contribute significantly to the swift progression of RP-ILD in patients with anti-MDA5-positive CADM. Due to the limited effectiveness of standard therapies, alternative treatment strategies are being investigated. Early detection and intervention are critical for enhancing outcomes in this complex condition. This study documents a case of anti-MDA5-positive CADM complicated by RP-ILD that was resistant to conventional treatment but showed a marked response to therapeutic plasma exchange (TPE).

## Case Report

**Figure 1:**
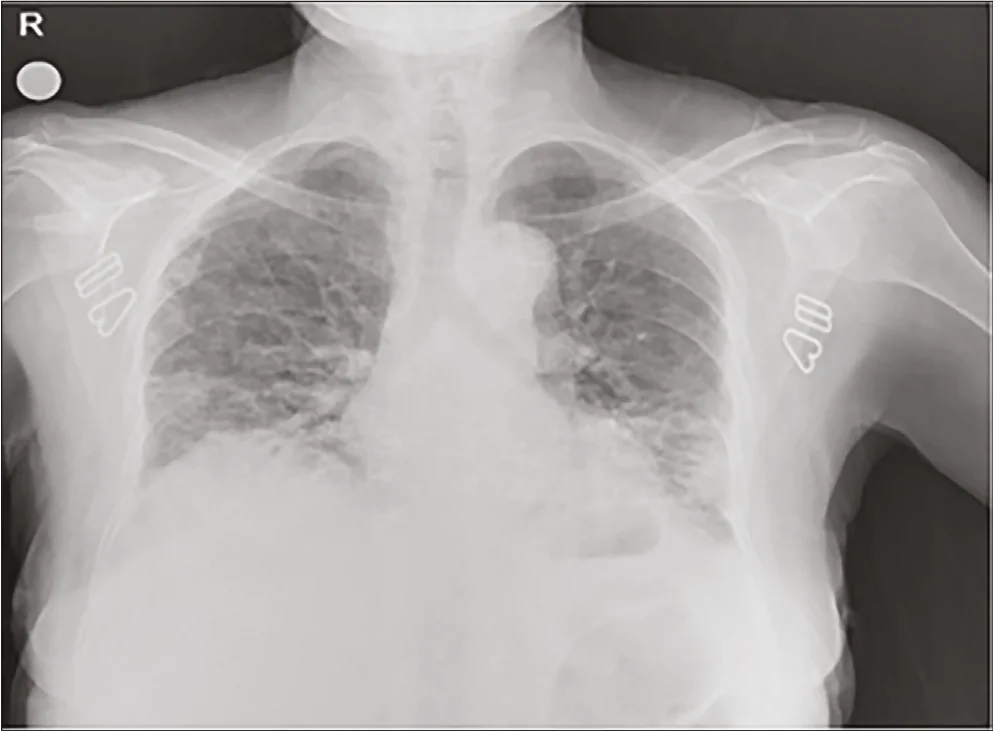
Chest X-ray before plasmapheresis.

**Figure 2:**
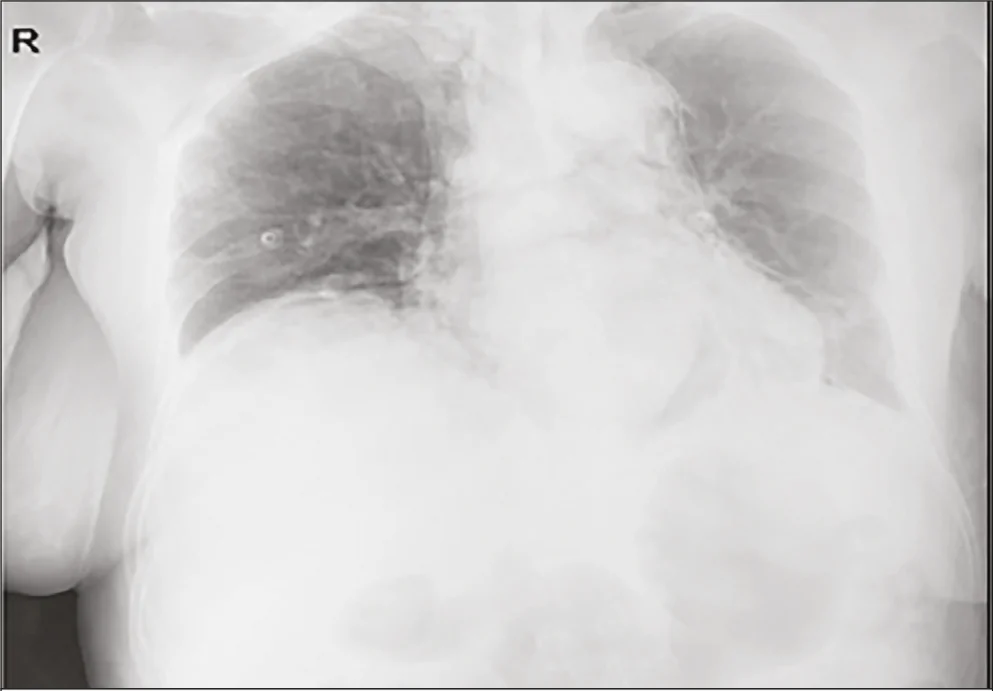
Chest X-ray after successful use of therapeutic plasma exchange.

A 60-year-old woman with a medical history of hypertension and an aneurysm presented with a three-week history of dry cough, worsening shortness of breath, and fatigue. Consent was obtained from the patient before reporting. She reported no muscle weakness or pain. Upon physical examination, findings included heliotrope rash, Gottron papules, periungual telangiectasia, and digital ulcers; muscle strength remained intact. Pre-TPE lab results indicated positive anti-MDA5 antibodies (other myositis-specific antibodies were negative), a creatine kinase (CK) level of 1,412 U L^-1^, a LDH level of 361 U L^-1^, a serum ferritin level of 1,450 ng mL^-1^, and a C-reactive protein (CRP) level of 22 mg L^-1^. Arterial blood gas analysis revealed hypoxemia (PaO_2_: 45 mmHg). Her oxygen saturation ranged between 86% and 92% while using a high-flow nasal cannula (HFNC) at an FiO_2_ of 80% delivered at a flow rate of 60 L min^-1^ (Figure 1). Initial therapy consisted of pulse methylprednisolone administered daily for three days, followed by oral prednisolone (1 mg kg^-1^/day) combined with tacrolimus (3 mg/day) and intravenous immunoglobulin (IVIG) administered over five days (total dose 2 g kg^-1^). Despite these efforts, her condition continued to deteriorate alongside increasing oxygen requirements; gastroscopy revealed subcutaneous emphysema, and thoracic computed tomography scans showed septal thickening in the lower lobes, bilateral ground-glass opacities indicating consolidations, and late-stage “honeycombing” suggestive of fibrosis. TPE commenced on day 17 of her intensive care unit (ICU) stay; five sessions were conducted on alternate days, with approximately two liters (40 mL kg^-1^) exchanged per session, using fresh frozen plasma. Clinical improvement was observed following the second session when FiO_2_ requirements decreased, leading up to HFNC discontinuation on day 19 in favor of nasal cannula oxygen support instead-ultimately ceasing all supplemental oxygen entirely by day 22 when she transitioned from ICU care back onto general ward status on day 27 without complications arising from the TPE procedure itself. Post-TPE lab values recorded ferritin reduced to 878 ng mL^-1^, CRP down to 5 mg L^-1^, LDH fell below 290 U/L, CK lowered significantly reaching 31 U/L (Figure 2).

## Discussion

Anti-MDA5-positive dermatomyositis frequently correlates strongly with pulmonary involvement resulting in heightened mortality risks.^1, 2^ Notable prognostic biomarkers such as hyperferritinemia alongside elevated LDH levels, coupled with worsening respiratory metrics, serve as key indicators regarding overall severity faced throughout the course of the disease process.^5, 6^ In the context surrounding the current case represented here significant drops identified within both ferritin and CRP values coincided directly alongside observable clinical improvements, thereby reinforcing the therapeutic advantages yielded through application implementing TPE procedures undertaken upon patients exhibiting refractory responses towards established first-line therapies typically employed comprising primarily either corticosteroid options or else calcineurin-based agents plus possible adjunct use of IVIG if deemed necessary.^3, 7^ However, refractory RP-ILD remains a major therapeutic challenge. TPE facilitates the rapid removal of circulating anti-MDA5 antibodies, immune complexes, and pro-inflammatory cytokines, which may explain the observed clinical stabilization in refractory cases.^8^ Previous case series and reviews have reported improved outcomes when TPE is initiated early.^9, 10^ This case adds to the growing body of evidence suggesting that TPE may serve as a life-saving bridge therapy in patients unresponsive to conventional immunosuppression. Emerging therapies, including Janus kinase inhibitors such as tofacitinib and baricitinib, have shown promise in improving lung function and survival in patients with RP-ILD who have failed standard treatments.^11^ Although these therapies remain under investigation, combining TPE with

## Conclusion

TPE may represent an urgent, life-saving measure for individuals with anti-MDA5-positive CADM complicated by RP-ILD when conventional treatments, such as corticosteroids or IVIG, are insufficient alone. Prospective and controlled studies are warranted to define the role of TPE in standard treatment protocols for this patient population.

## Ethics

**Informed Consent:** Consent was obtained from the patient before reporting.
